# Initial Weight Status Moderates the Impact of Exercise Referral Schemes on Improving Patients’ Body Mass Index

**DOI:** 10.1177/08901171261419478

**Published:** 2026-01-22

**Authors:** Carson Halliwell, Renaud Tremblay, Jonathon R. Fowles, Ryan E. R. Reid, Rebecca Moyer, Taniya S. Nagpal, Myles W. O’Brien

**Affiliations:** 1 School of Physiotherapy, Faculty of Health, 3688 Dalhousie University, Halifax, NS, Canada; 2 Centre de Formation Médicale du Nouveau-Brunswick, Moncton, NB, Canada; 3 Department of Medicine, Faculty of Medicine and Health Science, 7321 Sherbrooke University, Sherbrooke, QC, Canada; 4 Faculté des Sciences de l’Activité Physique, 7321 Université de Sherbrooke, Sherbrooke, QC, Canada; 5 Centre de recherche sur le vieillissement, CIUSSS de l’Estrie-CHUS, Sherbrooke, QC, Canada; 6 School of Kinesiology, 8689 Acadia University, Wolfville, NS, Canada; 7 Department of Human Kinetics, 1270 St Francis Xavier University, Antigonish, NS, Canada; 8 Faculty of Kinesiology, Sport, and Recreation, 3158 University of Alberta, Edmonton, AB, Canada

**Keywords:** body mass index, obesity management, exercise

## Abstract

**Purpose:**

To determine whether baseline body mass index (BMI) moderates BMI changes following completion of an exercise referral scheme (ERS).

**Design:**

Retrospective analysis.

**Setting:**

Data from the National ReferAll Database.

**Sample:**

634 adults out of 39,283 (64% female; mean age 54 ± 15 years).

**Intervention:**

Participants completed a community-based ERS involving physical activity counseling and supervised exercise.

**Measures:**

BMI was calculated as weight (kg) divided by height (m^2^) and participants were categorized by BMI: normal weight (<25.0 kg/m^2^), overweight (25.0-29.9 kg/m^2^), and obesity classes I (30.0-34.9 kg/m^2^), II (35.0-39.9 kg/m^2^), and III (≥40.0 kg/m^2^).

**Analysis:**

A 5 × 2 mixed methods analysis of covariance, adjusted for age, sex, ERS type, and follow-up duration examined BMI changes over time by group. Linear regression assessed the relation between baseline BMI and BMI change.

**Results:**

Baseline BMI predicted BMI reduction (β = −0.07 ± 0.01, 95% CI: −0.09 to −0.04, *P* < .001). Compared to the normal weight group, individuals with obesity classes I–III had significantly greater reductions in BMI (mean difference range: 0.83-1.01 kg/m^2^, all *P* < .001). No significant difference was observed between normal weight and overweight participants (*P* = .207).

**Conclusion:**

This study provides evidence that ERS can be effective in improving BMI outcomes among individuals who have an elevated initial BMI. These findings support the continued development and refinement of ERS as a scalable public health strategy to promote weight-related improvements.

## Purpose

Obesity continues to be a health concern worldwide. In Canada, nearly two-thirds of adults have an elevated body mass index (BMI) classified as obesity.^1^ Obesity is an independent risk factor for several co-morbidities including cardiovascular diseases, type 2 diabetes mellitus, musculoskeletal disorders, and premature mortality.^2,3^ Given these wide-ranging impacts, obesity represents a critical health concern with consequences at both the individual and healthcare system levels. In fact, the World Obesity Federation has estimated that by 2030 more than half of the world will have a BMI ≥25.0 kg/m^2^ and this will significantly impact healthcare costs upwards of $4.3 trillion (USD).^4^

To better identify individuals at heightened risk of obesity-related comorbidities, various assessment tools have been developed: most notably, BMI.^5^ Despite several valid criticisms, BMI remains an accessible and useful screening tool as it is simple, rapid to calculate, and inexpensive.^6^ It also tends to have a high correlation with percentage of body fat, though this may vary based on certain factors, such as age and ethnicity.^6^ Lifestyle habits such as a well-balanced eating pattern and engaging in regular physical activity are well established tools for BMI reduction^7^ and exercise participation can provide added benefit.^8^

Exercise referral schemes (ERS) are a clinical method where primary care providers refer patients to exercise specialists for physical activity counseling or supervised exercise programs.^9^ A recent review of ERS effectiveness has documented that the impact of ERS on BMI was mixed.^10^ Notably, previous research using the National ReferAll Database concluded that while ERS led to reductions in BMI, the changes were not clinically meaningful.^11^ Since then, additional evidence has suggested that weight reduction is primarily dependent on initial weight status.^12,13^ Previous evidence supports that higher BMI values are associated with a greater likelihood of achieving a 5% weight loss while taking part in a public weight loss program.^14^ Further, the probability of achieving a 5% weight reduction was found to be 1-in-12 for individuals in normal weight and 1-in-6 for individuals with class III obesity.^13^ Logically, an individual with a normal weight or underweight BMI is unlikely to largely alter their BMI within the typical 12-week timeframe of an ERS, but whether ERS improve BMI in those who may enter programs with an elevated BMI (ie, grouped as overweight or obesity), is unclear.

This study investigated whether pre-ERS BMI status moderates the changes in BMI following the completion of an ERS using data from the National ReferAll Database. We hypothesized that individuals with higher BMI at the initial time of referral would experience the greatest reductions in BMI after completion of the referred exercise program.

## Methods

### Design

This study retrospective cohort analysis, utilized data from The National ReferAll Database,^15^ a longitudinal dataset capturing information on individuals participating in ERS across the United Kingdom.^15^

### Sample

Between September 2011 and March 2021, the database included 39,283 participants who were referred to an ERS and provided informed consent at one of nineteen participating sites.^15^ In total, 15,680 participants were reported to have completed their prescribed ERS, 1776 had valid baseline BMI data, 1051 had valid follow-up BMI data, and 634 participants had follow-up BMI data that was greater than 8-weeks from the start of their ERS. All data were handled in accordance with the Global Data Protection Regulations. Only anonymized data were handled by the research team. All participants provided informed consent prior to inclusion into the original study. All data from the ReferALL dataset is publicly available at https://osf.io/uzbw9/.

### Measures

Participants self-reported age and sex. BMI was calculated as weight (kg) divided by height squared (m^2^). Participants were categorized into standard BMI classifications: normal weight (<25.0 kg/m^2^), overweight (25.0-29.9 kg/m^2^), obesity class I (30.0-34.9 kg/m^2^), obesity class II (35.0-39.9 kg/m^2^), and obesity class III (≥40.0 kg/m^2^).^16^ Due to a low number of participants classified as underweight (n = 9, mean BMI: 17.5 ± 0.79 kg/m^2^), this group was pooled with the normal weight category for analysis.^16^

### Intervention

Nineteen ERS were included in the National Referral Database at the time of its formation.^15^ At each site, participants underwent an initial screening and pre-assessment before engaging in an exercise or physical activity-based intervention.^15^ Upon program completion, follow-up assessments were conducted. While additional ERS data exists within the broader ReferAll system, only those schemes where participants provided consent for data inclusion were incorporated into The National Referral Database. The database encompasses multiple ERS; however, detailed data on their specific delivery or program are unavailable.

### Analysis

Demographics and clinical characteristics were summarized using means and standard deviations or counts and percentages. One way analysis of variance models were used to determine differences in baseline participant demographics and clinical characteristics between BMI groups. A 5 × 2 (group by time) mixed methods analysis of covariance model, adjusted for age, sex, ERS type, and follow-up measurement time was used to assess group by time interactions and main effects for BMI scores between groups at baseline and post ERS completion. A multivariate linear regression was conducted to examine the relation between baseline BMI and subsequent change in BMI. Outputted unstandardized β represent BMI change for every 1 kg/m^2^ increase in baseline BMI. Each model was adjusted for age, sex, ERS type, and follow-up measurement time. An α = 0.05 was set to indicate statistical significance for all tests and between group effect sizes were also calculated and interpreted using Cohen’s *d*. Statistical analyses were completed using SPSS (Version 29.0, IBM Corp., Armonk, NY, USA).

## Results

A total of 634 individuals (64% female) with an age of 54 ± 15 years were included. The majority of ERS program type (97%) was exercise on referral. BMI category groups did not differ in age, sex, ERS type, or in length of the ERS (all, *P* > .092; Table 1). Baseline BMI was a significant predictor of BMI change over the course of the ERS (β = −0.07 ± 0.01, 95% CI [−0.09:−0.04], *P* < .001), such that individuals with higher starting BMI experienced greater reductions in BMI (Supplemental Table 1). Of those classified as having an overweight or obese BMI, 70 individuals (14%) moved down a BMI category, while 20 individuals (4%) moved up (see Supplemental Figure 1).

**Table 1.** Participant Demographics and Clinical Characteristics

	Normal weight n = 130	Overweight n = 212	Obesity I n = 178	Obesity II n = 71	Obesity III n = 43
Age (years)	54 ± 17	54 ± 15	53 ± 14	52 ± 15	50 ± 12
Sex, no. of females (%)	79 (61)	128 (60)	119 (66)	47 (66)	31 (72)
ERS length (days)	84 ± 0.5	84 ± 0.0	84 ± 0.5	84 ± 0.7	84 ± 0.0
ERS type, no. (%)					
Cancer rehab	1 (1)	2 (1)	0 (0)	0 (0)	0 (0)
Cardiac rehab	2 (2)	1 (1)	1 (1)	1 (1)	0 (0)
Exercise on referral	125 (95)	207 (96)	173 (96)	68 (96)	42 (98)
Pulmonary rehab	1 (1)	1 (1)	0 (0)	0 (0)	0 (0)
Specialist exercise on referral	1 (1)	0 (0)	3 (2)	0 (0)	2 (2)
Weight management	0 (0)	1 (1)	1 (1)	2 (3)	0 (0)
Follow-up measure (days)	115 ± 53	110 ± 51	121 ± 54	123 ± 81	118 ± 34
Baseline BMI (kg/m^2^)	22.1 ± 2.1	27.4 ± 1.4^a^	32.1 ± 1.4^b^	37.1 ± 1.5^c^	43.9 ± 2.9^c^
Follow-up BMI (kg/m^2^)	22.1 ± 2.1	27.2 ± 2.0^a^	31.4 ± 2.0^b^	36.0 ± 2.5^c^	42.8 ± 3.7^d^
Baseline weight (kg)	63.3 ± 9.1	78.3 ± 9.6^a^	89.8 ± 10.1^b^	104.9 ± 13.1^c^	121.2 ± 17.6^c^
Follow-up weight (kg)	63.5 ± 9.1	77.2 ± 9.7^a^	87.5 ± 10.6^b^	101.3 ± 13.4^c^	116.9 ± 15.1^c^
Weight change (%)	0.02 ± 4.8	1.27 ± 4.6	2.63 ± 4.3	2.57 ± 5.4	2.12 ± 4.5

Note. ERS, exercise referral scheme; BMI, body mass index.

Values represent means ± standard deviations, unless otherwise indicated.

^a^Indicates statistical difference from normal weight.

^b^Indicates statistical difference from normal weight and overweight.

^c^Indicates statistical difference from normal weight, overweight, and obesity class I.

^d^Indicates statistical difference from normal weight, overweight, obesity class I, and obesity class II.

In terms of weight changes, individuals with normal weight saw no significant difference in weight loss following ERS (mean difference: −0.10 ± 3.19 kg, 95%CI [−0.67:0.47], *P* = .728; % weight reduction: 0.02 ± 4.8%). Individuals who had an overweight BMI (mean difference: −1.03 ± 3.63 kg, 95%CI [−1.53:−0.52], *P* < .001; % weight reduction: 1.27 ± 4.6%), obesity class I (mean difference: −2.36 ± 3.82 kg, 95%CI [−2.94:−1.78], *P* < .001; % weight reduction: 2.63 ± 4.3%), obesity class II (mean difference: −2.76 ± 5.60 kg, 95%CI [−4.15:−1.36], *P* < .001; % weight reduction: 2.57 ± 5.4%), and obesity class III (mean difference: −2.73 ± 5.72 kg, 95%CI [−4.59:−0.88], *P* = .002; % weight reduction: 2.12 ± 4.5%) all had reductions in weight following the ERS. After covarying for age, sex, ERS type, and follow-up measurement time, individuals in obesity classes I, II, and III groups had larger reductions in BMI over the ERS compared to individuals who were grouped as overweight or normal weight (Interaction effect: *P* < .001; Figure 1). Compared to individuals who had a normal weight or overweight BMI, individuals in all 3 obesity classes exhibited a greater reduction in BMI (all *P* < .004, all d > 0.38; Figure 1). Individuals who had an overweight BMI had no difference in BMI change compared to individuals who were in the normal weight category following the ERS (mean difference: 0.22 ± 0.17 kg/m^2^, 95% CI [−0.12:0.57], *P* = .207, d = 0.15).

**Figure 1.**
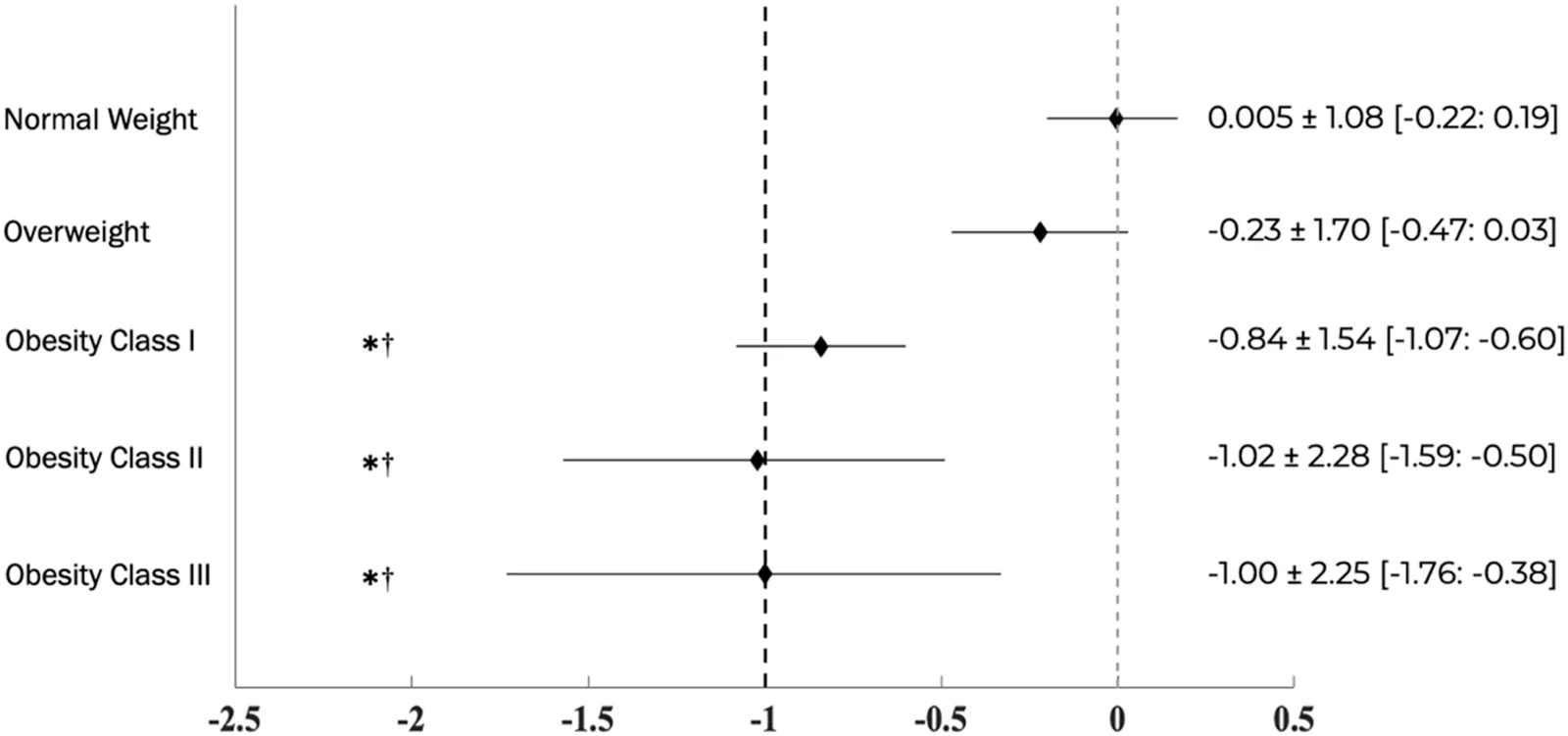
Forest plot indicating the mean difference and 95% bootstrapped confidence intervals between baseline and follow-up, in body mass index for each body mass index category. The dashed black line represents a clinically important improvement in body mass index. *Indicates significant difference in BMI change compared to normal weight. ^†^Indicates significant difference in BMI change compared to overweight

## Discussion

The results support our hypothesis that ERS are most effective for individuals with higher baseline BMI, particularly amongst those who would be in the obesity category. Despite conflicting reports on whether ERS impacts BMI,^10^ we provide clarity in that only those with pre-ERS obesity, as defined by BMI, exhibit reductions. However, given the absence of a non-ERS comparison group, our findings should be viewed as observational evidence of BMI change in routine ERS practice rather than causal estimates of program effectiveness.

Unlike traditional exercise interventions, which often recruit highly motivated individuals in tightly controlled environments, ERS programs are delivered in routine care and are designed to be accessible to diverse populations with varying health status.^11^ In this study, individuals classified as having obesity experienced greater reductions in BMI compared to those in the overweight or normal weight categories, even after adjusting for age, sex, ERS type, and follow-up duration. These changes were statistically significant, with reductions approaching or exceeding 1 kg/m^2^, or approximately 2.5% weight reduction. These amounts have been associated with a 16% reduction in the odds of type 2 diabetes mellitus^17^ and corresponding decreases in blood pressure of −1.05 mm Hg (95% CI, −1.43:−0.66) systolic and −0.92 mm Hg (95% CI, −1.28:−0.55) diastolic per kilogram of weight loss,^18^ reflecting meaningful improvements in metabolic risk and long-term health outcomes.^19^ Importantly, participants who were excluded from analyses due to not having a valid follow-up BMI measure were, on average, approximately 2 kg/m^2^ heavier at baseline than those included therefore, the current results may underestimate the true effect of the ERS program on individuals with higher BMI (Supplemental Table 2). While regression to the mean may partly contribute to the greater BMI reductions among individuals with obesity, the persistence of these associations after adjustment suggests it is unlikely to fully explain the observed pattern.

A previous analysis of the ReferALL database observed that ERS led to a statistically significant but clinically negligible change in BMI,^11^ likely masked by grouping all participants together.^11^ To address this, we stratified participants by BMI category to assess group-specific ERS effects. The results support that effectiveness appears dependent on baseline BMI, aligning with Wilder’s Law of Initial Value, which posits that pre-treatment levels influence treatment response.^20^ In our study, each 5 kg/m^2^ higher baseline BMI corresponded to an additional 0.33 kg/m^2^ reduction. However, it is important to note that BMI does not accurately measure clinical obesity, defined as excess or dysfunctional adipose tissue that impairs health.^7^ While BMI is a widely used indicator of adiposity, it does not distinguish between fat and lean mass and may therefore underestimate changes resulting from exercise-based interventions.^7^ Measures such as waist circumference or body composition (eg, fat mass, lean mass, or visceral adiposity) may respond more sensitively to ERS participation, particularly when improvements reflect increased muscle mass or reduced central adiposity rather than overall weight loss.^21^ Obesity management guidelines emphasize that BMI may be used as a screening tool for obesity or a population level assessment.^7^ Future research should also consider the effectiveness of ERS on comprehensive and patient-centred measures of obesity as recommended in recent obesity management guidelines (eg, Edmonton Obesity Staging System, Clinical Obesity, King’s Obesity Staging Criteria).^21^

Importantly, not having information on the nature, intensity, and adherence to the ERS, may have contributed to variability in outcomes. Evidence from previous reviews indicates that ERS delivered by a qualified exercise professional and incorporating activity counselling and/or structured exercise programming produce more favourable effects on both self-reported physical activity and objectively measured aerobic fitness.^9^ Therefore, it is plausible that participants who engaged in more structured or professionally guided ERS experienced greater BMI improvements than those in less intensive or inconsistently delivered programs. Future research should aim to capture detailed information on ERS delivery characteristics and participant adherence to clarify how program components influence outcomes.

A key strength of this study is the use of data from the National ReferAll Database, which captures real-world outcomes from a large, diverse sample of individuals participating in ERS across the United Kingdom. Our stratified analysis by baseline BMI category provides a more nuanced understanding of ERS effectiveness, helping to clarify prior mixed results by addressing the heterogeneity of participant characteristics. However, limitations should be acknowledged. First, the dataset includes only participants with valid follow-up BMI data, potentially introducing selection bias, as those who did not have follow-up may have differing outcomes. Individuals who did not have valid follow-up had a higher baseline BMI compared to those included (mean difference: 2.34 ± 0.22 kg/m^2^, 95% CI [1.96:2.77], *P* < .001, d = 0.34). Information regarding why patients may have dropped out was not available and this may be an important direction for future research. The lack of detailed information on program content and exercise prescription limits the ability to determine how specific exercises contributed to BMI changes. Additionally, we were constrained by the variables available within the ReferAll Database, which did not include covariates such as comorbidities, medication use, session attendance, socioeconomic measures, or nutritional interventions. The absence of these data limits our capacity to account for potential confounders that could influence both BMI and participation in the ERS. Additionally, we were limited by the data available within the ReferAll Database, which restricted our ability to examine other potentially important outcomes such as waist circumference.

This study provides evidence that ERS can be effective in improving BMI outcomes among individuals who have an elevated initial BMI. These findings support the continued development and refinement of ERS as a scalable public health strategy to promote weight-related improvements.

**So What?**

**What Is Already Known on This Topic?**

Exercise referral schemes are commonly used in routine healthcare to encourage physical activity and support weight management. However, previous research has shown mixed results regarding their impact on body mass index, especially when differences in starting weight are not considered.

**What Does This Article Add?**

This study suggests that individuals with higher initial body mass index tend to experience greater reductions in body mass index following participation in an exercise referral scheme. In contrast, those in normal or overweight categories appear to show smaller or negligible changes, which may help explain inconsistencies reported in previous studies.

**What Are the Implications for Health Promotion Practice or Research?**

Findings provide support that exercise referral schemes may be particularly beneficial for individuals with higher baseline body mass index. Health promotion initiatives could consider accounting for baseline body mass index when evaluating program outcomes.

## Supplemental Material


Supplemental Material - Initial Weight Status Moderates the Impact of Exercise Referral Schemes on Improving Patients’ Body Mass Index
Supplemental Material for Initial Weight Status Moderates the Impact of Exercise Referral Schemes on Improving Patients’ Body Mass Index by Carson Halliwell, Renaud Tremblay, Jonathon Fowles, Ryan E. R. Reid, Rebecca Moyer, Taniya S. Nagpal, and Myles W. O’Brien in American Journal of Health Promotion.

## Data Availability

The data used for analyses in this paper are publicly available at https://osf.io/uzbw9/.
