# Supplementary material for: Initial Weight Status Moderates the Impact of Exercise Referral Schemes on Improving Patients’ Body Mass Index
Source: Am J Health Promot. 2026 Jan 22;40(8):941–6. doi: 10.1177/08901171261419478 (PMC13646416; doi:10.1177/08901171261419478)
Supplement: Supplementary file 1 [file sj-pdf-1-ahp-10.1177_08901171261419478.pdf]

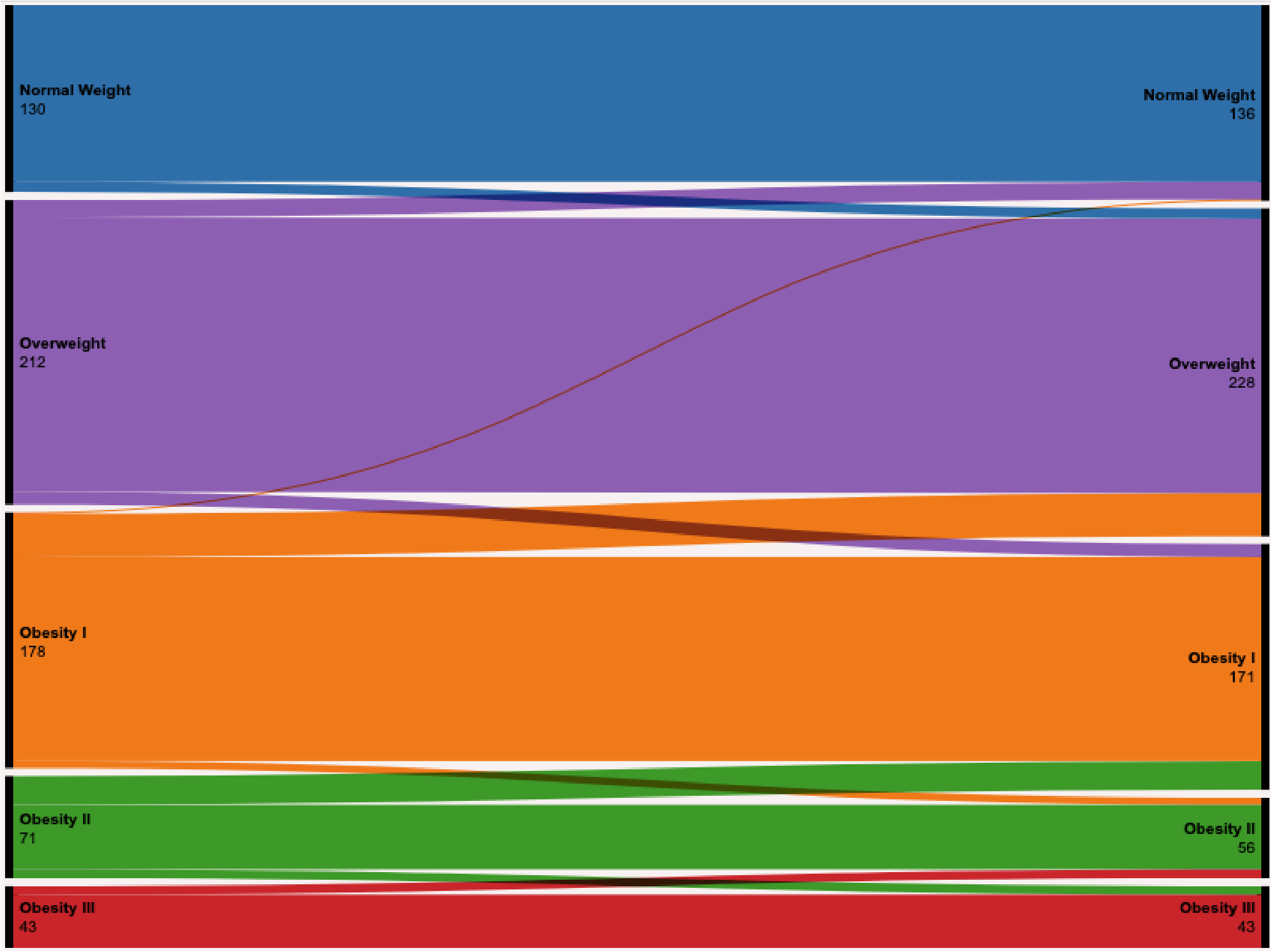

Supplemental Figure 1. Alluvial demonstrating changes in body mass index categories before and after the exercise referral scheme.

Supplemental Table 1. Multivariate linear regression output examining the association between age, sex, exercise referral scheme type and baseline body mass index on subsequent body mass index change following the exercise referral scheme.

Variables	β	Standard Error	95% CI	P-value	R^2
Overall Model	-	-	-	**<0.001**	**0.06**
Age	-0.003	0.005	-0.012: 0.006	0.545	-
Sex	0.038	0.146	-0.248: 0.324	0.794	-
ERS Type	0.001	0.001	-0.001: 0.003	0.100	-
Baseline Body Mass Index	**-0.065**	**0.012**	**-0.088: -0.042**	**<0.001**	**-**

Data are presented as unstandardized β, standard error, and 95% confidence intervals (95% CI). Bolded values indicate statistical significance at $p < 0.05$. R^2 represents the adjusted R^2 value.
Note. ERS = Exercise referral scheme

Supplemental Table 2. Baseline demographic comparison between individuals included in the study and those without valid follow-up data

	Included n=634	Excluded n=1104
Age (years)	54 ± 15	53 ± 16
Sex, no. of females (%)	404 (64)	688 (62)
ERS Type, no. (%)		
Cancer Rehab	3 (0.5)	5 (0.5)
Cardiac Rehab	5 (0.8)	20 (2)
Exercise on Referral	615 (97)	1063 (96)
Pulmonary Rehab	2 (0.3)	2 (0.2)
Specialist Exercise on Referral	6 (0.9)	5 (0.5)
Weight Management	4 (0.6)	1 (0.1)
Stroke Rehab	-	8 (0.7)
Baseline BMI (kg/m^2)	29.8 ± 6.1	32.1 ± 6.4*
Baseline BMI Categories, no. (%)		
Normal Weight	130 (21)	222 (20)
Over Weight	212 (33)	337 (31)
Obesity Class I	178 (28)	273 (25)
Obesity Class II	71 (11)	169 (15)
Obesity Class III	43 (7)	103 (9)
Baseline Weight (kg)	84.2 ± 18.8	87.9 ± 20.6*

Values represent means ± standard deviations, unless otherwise indicated.
* Indicates statistical difference between groups
Note: ERS = Exercise referral scheme; BMI = Body mass index
